# Editorial: Optimizing surgical procedures in renal cancers to improve patient outcomes

**DOI:** 10.3389/fonc.2022.1019946

**Published:** 2022-09-27

**Authors:** Hiten D. Patel, Arnav Srivastava

**Affiliations:** ^1^ Department of Urology, Feinberg School of Medicine, Northwestern University, Chicago, IL, United States; ^2^ Section of Urologic Oncology, Rutgers Cancer Institute of New Jersey, New Brunswick, NJ, United States

**Keywords:** kidney cancer, surgery, renal cell carcinoma, outcomes - health care, nephrectomy, radical nephrectomy, partial nephrectomy, robotic surgery

Clinical stage migration led to the diagnosis of smaller renal tumors over a period of several decades; however, the stage distribution of renal cell carcinoma (RCC) has now remained relatively stable or 15 years in the Untied States and likely in other countries where use of cross-sectional imaging is pervasive (1). Minimally-invasive approaches and a push for nephron-sparing procedures has decreased morbidity related to perioperative outcomes and development of surgically induced chronic kidney disease (2, 3). The present Research Topic collection, Optimizing Surgical Procedures in Renal Cancers to Improve Patient Outcomes, includes several relevant review articles and original studies which highlight the potential role of some of these advances and future directions for optimizing surgical management of RCC.

Radical nephrectomy is the historic gold standard for the treatment of localized RCC with the laparoscopic approach demonstrating equivalent oncologic control but improved perioperative outcomes over the open approach (4). Since the introduction of robotics, robotic-assisted radical nephrectomy (RARN) has taken over some of the market share from both laparoscopic radical nephrectomy (LRN) and the open approach. Li et al. performed a systematic review of the literature and meta-analysis showing that perioperative outcomes were no different for RARN compared to LRN in a pooled population of 1,832 patients. The findings are important as they provide further evidence that the choice of RARN over LRN is not justified by superiority of either approach. Rather, the pressure to utilize purchased robots, surgeon preference, and patient demand from marketing led to the migration of procedures toward RARN. Costs are another concern given the increased operating room and supply costs for RARN (5, 6). However, there may be cases where RARN enables successful performance of a minimally-invasive approach as an alternative to open radical nephrectomy where a surgeon would not have been comfortable attempting LRN (7, 8).

Although oncologic control for RARN and LRN raised few concerns over open radical nephrectomy, the push toward minimally-invasive nephron-sparing surgery for amenable tumors added additional questions given the lack of tactile feedback and potential risk for a positive surgical margin (PSM). You et al. perform a systematic review and meta-analysis demonstrating laparoscopic partial nephrectomy (LPN) had similar recurrence and cancer-specific survival rates but with improved perioperative outcomes relative to open partial nephrectomy. The findings are notable given the breath of cohort studies to date, but they also found that PSM rates were slightly higher for LPN in the overall sample although rates for cT1a patients were no different. Notably, while the lower increase in serum creatinine for LPN is to be interpreted with caution, a recent randomized trial from Brazil totaling 208 patients suggested the same result of better kidney function preservation for LPN (9).

The migration toward nephron-sparing surgery may also have implications for pediatric renal tumors; a meta-analysis by Chen et al. suggests nephron-sparing surgery could be considered for Wilms tumor although the sample was small with most of the 297 patients in the nephron-sparing group coming from the SIOP (International Society of Pediatric Oncology) experience or data from SEER (10). Currently, the Children’s Oncology Group suggests use of nephron-sparing surgery in management of bilateral tumors or unilateral tumors <4cm in size if deterioration of renal function may be expected.

When considering nephron-sparing options, tumor complexity is a critical piece of the decision process along with other clinical and patient factors. The relative risks of partial nephrectomy increase relative to other options (i.e. active surveillance, thermal ablation, and radical nephrectomy) when operating on more complex tumors. As a novel option to other well known nephrometry scores to quantity complexity, Zhang et al. describe the “3S+f” system which includes components such as size, site (location – lateral/upper vs. medial/lower), and side (distance to hilum) which are represented in the RENAL and PADUA systems. They also include evaluation of perinephric fat rated from 0 to 3 points ranging from none to sticky/thick on cross-sectional imaging. The association of “3S+f” was equivalent to RENAL and PADUA for predicting perioperative outcomes.

In another study, Jiang et al. focus on complex renal tumors where perioperative outcomes for minimally-invasive partial nephrectomy may be of highest concern; they perform a review evaluating application of 3D printing technology to minimally-invasive partial nephrectomy [LPN or robotic-assisted LPN (RALPN)] for complex renal tumors finding that 3D-preoperative assessment was associated with shorter operative time, shorter warm ischemia time, less blood loss, better preservation of renal function, and fewer complications. An additional novel option during RALPN for complex tumors is described by Bai et al. where they applied a renal artery balloon catheter to feasibly achieve cold ischemia by perfusing lactated Ringer’s solution.

Over time, it has become clear that renal function after partial nephrectomy is more a function of preserved kidney parenchymal mass than of warm ischemia time when the latter is not unduly prolonged (11). However, consideration of tumor enucleation over a standard margin approach and avoidance of suture renorrhaphy are additional factors that may optimize postoperative renal function (12, 13). In a systematic review and meta-analysis, Xu et al. report on 13 studies suggesting improved perioperative outcomes and better preservation of renal function with tumor enucleation over standard margin partial nephrectomy without compromising oncologic outcomes. By leveraging the tumor pseudocapsule, parenchymal preservation is maximized with tumor enucleation. The reduced trauma induced by tumor enucleation also enables consideration of avoiding a suture renography. In a propensity score matched analysis, Zhang et al. reported their experience with conventional vs. sutureless LPN performed *via* an enucleation technique; they found avoidance of suture renography was associated with less decrease in eGFR.

We have made great progress in techniques, technology, and understanding over the past two decades to improve outcomes for patients receiving surgery for renal tumors. The present collection highlights some of the progress and trends in management over time. Future directions include evaluating surgical approaches and timing of surgery for high risk and locally advanced RCC as options for perioperative systemic therapy continue to evolve and a reassessment of the role of surgery for metastatic RCC in the era of immuno-oncology agents​​ (14–17).

## Author contributions

HP and AS drafted and critically revised the manuscript. All authors contributed to the article and approved the submitted version.

## Conflict of interest

The authors declare that the research was conducted in the absence of any commercial or financial relationships that could be construed as a potential conflict of interest.

## Publisher’s note

All claims expressed in this article are solely those of the authors and do not necessarily represent those of their affiliated organizations, or those of the publisher, the editors and the reviewers. Any product that may be evaluated in this article, or claim that may be made by its manufacturer, is not guaranteed or endorsed by the publisher.
